# Continuous depth profile of the rock strength in the Nankai accretionary prism based on drilling performance parameters

**DOI:** 10.1038/s41598-018-20870-8

**Published:** 2018-02-14

**Authors:** Yohei Hamada, Manami Kitamura, Yasuhiro Yamada, Yoshinori Sanada, Takamitsu Sugihara, Saneatsu Saito, Kyaw Moe, Takehiro Hirose

**Affiliations:** 1 Kochi Institute for Core Sample Research, Japan Agency for Marine-Earth Science and Technology, 200 Monobe-otsu, Nankoku, Kochi, 783-8502 Japan; 2 Department of Earth and Planetary Systems Science, Graduate School of Science, Hiroshima University, 1-3-1 Kagami-yama, Higashi-Hiroshima, 739-8526 Japan; 3 Research and Development Center for Ocean Drilling Science, Japan Agency for Marine-Earth Science and Technology, 3173-25, Showa-machi, Kanazawa-ku, Yokohama-city, Kanagawa 236-0001 Japan; 4 The Center for Deep Earth Exploration, Japan Agency for Marine-Earth Science and Technology, 3173-25, Showa-machi, Kanazawa-ku, Yokohama-city, Kanagawa 236-0001 Japan; 5 Present Address: Geological Survey of Japan, National Institute of Advanced Industrial Science and Technology (AIST), 1-1-1 Higashi, Tsukuba, 305-8567 Japan

## Abstract

A new method for evaluating the *in situ* rock strength beneath the seafloor is proposed and applied to the Nankai Trough accretionary prism. The depth-continuous *in situ* rock strength is a critical parameter for numerous studies in earth science, particularly for seismology and tectonics at plate convergence zones; yet, measurements are limited owing to a lack of drilled cores. Here, we propose a new indicator of strength, the equivalent strength (EST), which is determined only by drilling performance parameters such as drill string rotational torque, bit depth, and string rotational speed. A continuous depth profile of EST was drawn from 0 to 3000 m below the seafloor (mbsf) across the forearc basin and accretionary prism in the Nankai Trough. The EST did not show a significant increase around the forearc basin–accretionary prism boundary, but it did show a clear increase within the prism, ca. below 1500 mbsf. This result may indicate that even the shallow accretionary prism has been strengthened by horizontal compression derived from plate subduction. The EST is a potential parameter to continuously evaluate the *in situ* rock strength during drilling, and its accuracy of the absolute value can be improved by combining with laboratory drilling experiments.

## Introduction

The *in situ* continuous strength of the crust is one of the most fundamental and vital information in various research fields of earth sciences. In geological and geophysical research in subduction zones in particular, the rock strength is necessary for evaluating development mechanisms of accretionary prism/forearc basins^1,2^, spatiotemporal variations in the stress state^3–5^, and fault reactivation potentials (slip tendency)^6,7^. The triaxial compression experiment is a common approach to measuring the strength of rock core materials recovered by deep-sea drilling^8,9^. Assumptions of the *in situ* stress conditions, however, are necessary for experimental strength measurements. As the experiments depend on core quality and availability, the experimentally determined strength data is intermittent and its continuous profile cannot be visualised. Even at the Nankai Trough, the most studied plate subduction zone by the Integrated Ocean Drilling Program (IODP)^10^, we were forced to utilise either the strength indirectly estimated through the empirical correlation between the strength and P-wave velocity or the experimental strength from limited cores^11–13^. A similar situation also arises in most offshore drilling sciences^14^. Despite its importance, the depth profile of *in situ* strength has been practically left unknown. Therefore, we propose a method for the direct and continuous measurement of the relative *in situ* rock strength using only drilling performance parameters. Drilling parameters can be easily obtained from any drill hole, even in non-coring and non-logging operations or in challenging environments.

Evaluation of the formation rock properties from drilling parameters such as weight on bit (WOB) and drill string rotational torque (Tr) was first proposed by Teal (1964)^15^ and denoted the “specific energy” (SE). SE is used mainly for drilling optimisation in resource industries and has been further developed into the mechanical specific energy^16,17^. Several data conversion techniques have also been proposed to evaluate the *in situ* rock strength during drilling with various types of bits, such as roller-cone bits^18,19^ and fixed-cutter bits^20–24^. Recently, in a scientific deep-sea drilling operation, the evaluation of the apparent rock frictional strength was attempted based on WOB and Tr^23^. These researchers discussed the relationship between the rock strength and drilling parameters, considering the bit shape and input energy, using highly accurate and well-stabilised drilling parameters^23,24^. On the other hand, during typical offshore drilling operations, the recorded drilling data are usually disturbed by sea undulation. Additionally, large frictional forces can occur between the seawater or borehole wall and the drill string pipe, rather than the formation rock at the drill bit. Thus, if an on-bottom measurement-while-drilling system is not implemented, the above methods cannot be applied in typical deep-sea drilling operations^25^.

Here, we define a background-removed mechanical parameter, the equivalent strength (EST), derived from only Tr, bit depth, and pipe rotation speed measured at the surface of the rig during drilling. The proposed method was applied to drilling data obtained from IODP Site C0002 at the Nankai Trough, which is the main site for deep drilling to the seismogenic zone, to evaluate the continuous *in situ* strength in the Nankai accretionary prism. This method allows estimation of the strength without assuming uncertain *in situ* conditions (e.g., stress and fluid pressure conditions) or using special techniques. The continuous strength was also evaluated by applying previously obtained drilling parameters, which enabled us to clarify the strength profile extracted from the limited experimental strength data.

## Results

### Drilling parameters at IODP Site C0002 in the Nankai Trough

**Figure 1:**
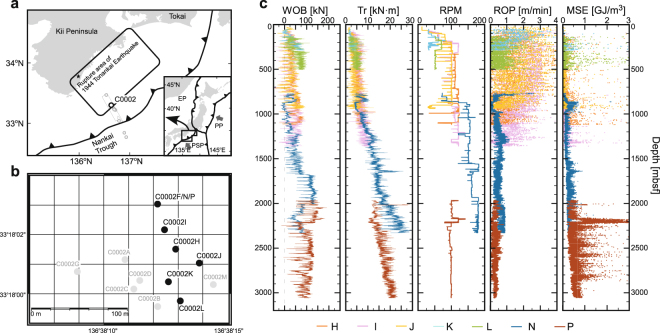
Locations of the NanTroSEIZE drilling sites (**a**) and holes (**b**) and an example of the drilling performance parameters collected at site C0002 (c). (**a**) Regional tectonic setting of the IODP NanTroSEIZE drilling transect and location of site C0002^10,48^ (modified from Hamada *et al*.^49^). The black outline in the map shows the region from which 3D seismic data was obtained by NanTroSEIZE. Abbreviations in the inset include PP, Pacific Plate; PSP, Philippine Sea Plate; and EP, Eurasian Plate. (**b**) Magnified map of the holes drilled at site C0002. The drilling parameters recorded in the holes are shown as black dots. **c**: Basic drilling parameters recorded at site C0002. Each colour indicates the hole from which the data were acquired. Abbreviations in the inset include WOB, weight on bit; Tr, torque; RPM, rotations per minute; ROP, rate of penetration; and MSE, mechanical specific energy.

The Nankai Trough is a major subduction zone where the Philippine Sea Plate is subducting beneath the Eurasian Plate at a rate of 4.1–6.5 cm/year^26^ (Fig. 1a). The IODP Nankai Trough Seismogenic Zone Experiment (NanTroSEIZE) Expeditions 314, 315, 332, 338, and 348 drilled 14 holes in Site C0002 from the forearc basin to the accretionary prism in order to reach the hanging wall of the plate boundary fault and the fault itself^6^ (Fig. 1b). Shallow non-riser drillings for both coring and logging-while-drilling (LWD) were conducted mostly in the forearc basin to investigate the physical properties, structure, and stress state. The riser drilling method was also applied in the vicinity of non-riser holes to penetrate the accretionary prism beneath the forearc basin (holes F, N, and P), and this method will be used to penetrate the megathrust in the future. Core samples and logging data collected from these holes show that the drilled formations (lithofacies) are consistent with each hole; the forearc basin consists of greenish-grey silty sandstone (0–975.5 mbsf), while the accretionary prism is composed mainly of greenish-grey silty claystone and fine silty claystone (975.5 mbsf)^27^. From eight holes (F, H, I, J, K, L, N, and P) drilled at Site C0002, basic drilling parameters, such as weight on bit (WOB), top drive torque, and bit depth, were recorded at the surface, and seven of these holes (excluding Hole F) were targeted in this study (Fig. 1c). The data can be accessed or requested at http://sio7.jamstec.go.jp/. Although WOB was not well stabilised at all holes, possibly because of rough seas, the rotations per minute (RPM) values showed steady rotation, and Tr showed little fluctuation and tended to increase with depth. The mechanical specific energy (MSE) calculated from the above parameters^15,16^ also increased with depth from 0 to ~1 GJ/m^3^, with large variations. The hole properties and the drill assemblies used in the drillings are summarised in Table 1. Data from hole F cannot be applied in this research because an underreamer was used during downward drilling, which may have generated extra cutting resistance. As described above, the drilling methods (riser/non-riser, coring/LWD) and conditions were quite different among the various holes (Table 1). However, in this study, the EST can be evaluated for each hole using the equations described below (see Methods section and Supplementary Table S1).

**Table 1:** Detailed hole properties at site C0002.

Site C0002						
Hole	Bit size [in]	Top [mbsf]	Bottom [mbsf]	LWD	MWD	Riser
H	10–5/8	0.0	1120.5			
I	10–5/8	0.0	1360.3			
J	10–5/8	0.0	940.0			
K	11–7/16	0.0	286.5			
L	11–7/16	0.0	505.0			
N	17	860.3	2328.9		+	+
P	12–1/4	1936.8	3056.6	+	+	+

Bit size, top and bottom depths of the holes, and drilling types in each hole. Abbreviations in the inset include LWD, logging-while-drilling; and MWD, measurement-while-drilling.

### Depth profile of the EST at Site C0002

**Figure 2:**
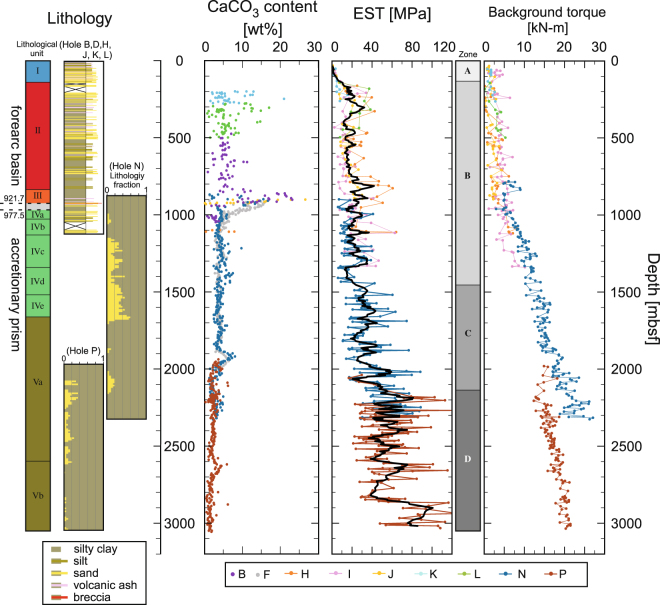
Depth profiles of lithology, calcium carbonate content, calculated formation strength, and drilling background torque. Lithological units and columnar sections were established based on both core and cutting samples. The percentage of silty claystone vs sandstone was measured by optical observations of the cuttings. Units I–III and the subjacent Units IV–V are interpreted as forearc basin sediment and shallow accretionary prism, respectively^27,29^. The boundary is identified between 921.7 mbsf (Hole B)^28^ and 1025.5 mbsf (Hole F)^27^. The calcium carbonate (CaCO_3_) content was obtained from measurements of the core and cutting samples. The converted strength and calculated background torque in this study are shown as coloured dots in the right-hand boxes. The black line on the dots is the running average of all holes within 5 data points. Each colour indicates the drill hole from which the data or samples were obtained. Note that not all of the drill holes used in the EST calculations are the same as the holes from which the CaCO_3_ and lithology data were acquired.

Figure 2 summarises composites of the lithologic data of the core and cuttings from the cruise reports^27,28^ as well as the calculated EST profile at Site C0002. The colours of the data indicate the different holes. The black line in the centre column is the running average of 5 points of aggregated data from different holes. The EST profile at all holes increases gradually with depth. The values are almost 0 MPa at the surface and 40–100 MPa at the bottom of the holes. We defined the mechanical zones (Zones A–D) based on the EST, independent of the lithological unit, to characterise the depth profiles of the EST. Zone A (0–132.3 mbsf) is the uppermost portion of the holes. The EST increases gradually to ~1 MPa by 77.6 mbsf and then increases suddenly to 6.2 MPa at the bottom of Zone A. Zone B (132.3–1458.5 mbsf) is characterised by a constant baseline for the EST of ~20 MPa. In this zone, the mean values of the EST do not show a clear change, though some positive peaks appear at 150–400 mbsf and 800–1000 mbsf. In Zone C (1442.5–2141.5 mbsf), the EST profile shows an overall steep increasing trend with larger fluctuations than in the upper zones. The amplitude of the fluctuation reaches ~39.8 MPa at around 2000–2100 mbsf, even in the running-averaged values. The EST reaches 53.7 MPa at the bottom of Zone C. In Zone D (2141.5–3011.5 mbsf; the lowest part of the holes), the mean value of the EST again tends to be constant at 2850.0 mbsf but then jumps to ~100 MPa at ~2900 mbsf. Note that the described mechanical zones do not correspond to the lithologically defined units (Fig. 2), except for Zone A, which is approximately consistent with Unit I. A major lithological boundary of unconformity between the forearc basin and accretionary prism was reported at 921.7–1025.5 mbsf^27–29^. However, the EST profile does not indicate any significant change or gap in that interval but shows a steeper baseline from around 1500 mbsf. In contrast to the large variation in the EST, the background torque shows a smooth increase from 0 to ~20 kN-m from top to bottom (Fig. 2). The slope of the background torque in Hole N is steeper than in the others, which corresponds to higher torque and RPM values (Fig. 1).

## Discussion

An attempt to extract the formation mechanical properties from the drilling parameters was made as described above^15,24^. Comparing the EST (Fig. 2) with the MSE (Fig. 1c), the EST shows relatively stable profiles and reasonable absolute values. Many of the deviated values or large profile gaps between holes in the MSE (e.g., 2200 mbsf) are not observed in the EST. This is because the background torque consumed by different types of resistance, such as torsion of the drill strings and friction between the pipe and borehole, is approximately subtracted from the measured torque during data processing, revealing the distinct formation properties. Large fluctuations can still be seen in the EST at the depth interval of 2270–2275 mbsf (Supplementary Fig. S3). In the EST calculation, we assumed that 5 m calculation intervals consist of homogeneous formation. This assumption is not entirely satisfactory, as a visible lithological boundary was observed in the logging data in this fluctuation interval (see Methods section).

**Figure 3:**
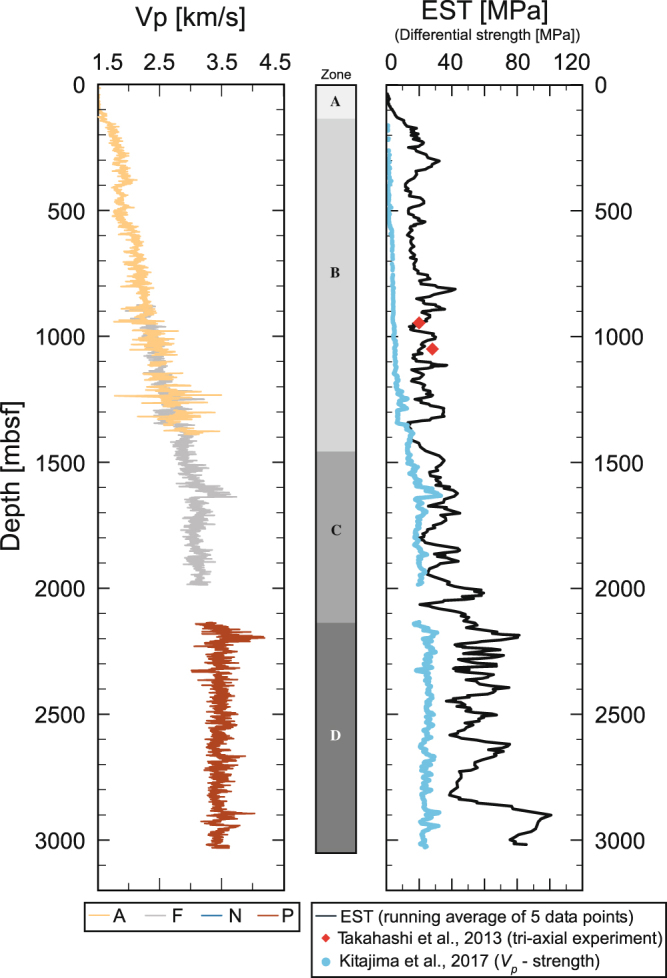
P-wave velocity at Site C0002 and strength profiles derived from the triaxial test, P-wave velocity, and drilling parameters. The depth profile of the P-wave velocity was acquired from logging-while-drilling in Holes A, F, N, and P^10,28,29^. Each line colour indicates the drill hole from which the logging data were obtained. The black EST line is the running average of all holes within 5 data points (Fig. 2). The red squares in the column are the measured strengths obtained in laboratory experiments^9^, and the light blue dots show the strengths estimated from the sonic velocity-porosity-strength relation^13^.

To examine the validity of the EST, we compared it with the downhole logging data of the P-wave velocity of formation^10,27–29^ (V_p_) and the strength data experimentally determined in previous studies^11,13^ (Fig. 3). V_p_ is known to be correlated with unconfined compressive strength (UCS)^30^. The V_p_ logging tool used at this site is located 6–27 m above the bit^10,27–29^. Although there is a time lag of several minutes to several hours between the drilling operation and V_p_ measurement, it is expected that V_p_ shows some correlation with strength. The general depth trends of the EST and V_p_ are consistent (Fig. 3). Both the EST and V_p_ gradually increase with depth up to ~2000 m in Zones A to C, and their baselines are almost constant, particularly in Zone D. At smaller scale depth intervals (e.g., several metres in Fig. S3), the EST correlates with local fluctuations in the V_p_ data much more so than at larger scales, as shown in Fig. 3. All of the peaks in the V_p_ data do not correspond to peaks in the EST. This is because the data sampling intervals are different for the EST calculations and V_p_ measurements: the former is approximately 5 m, while the latter is 15 cm (see Methods section).

The core sample strengths under *in situ* conditions determined by triaxial compression experiments^9^ are consistent with the EST (red squares in Fig. 3). The experiments were conducted on intact specimens, assuming that the pore pressure is hydrostatic and the vertical stress is the minimum principal stress (i.e., reverse-fault stress regime)^11^. However, the current accretionary prism lies in a strike-slip faulting regime^5,12^. Therefore, the experimental strength may be overestimated compared to the actual *in situ* strength, suggesting that the EST may also be overestimated.

On the other hand, the strength estimated from the empirical V_p_-porosity-strength relation^13^ shows smaller values compared with the EST (blue dots in Fig. 3). Kitajima *et al*.^13^ first constructed the relationship between porosity and V_p_. They converted the porosity measured from core samples into the *in situ* porosity using the recompression index values determined by consolidation experiments on samples recovered from 553–2018 mbsf^31–33^. Extrapolating the porosity-V_p_ relation, they defined the *in situ* porosity from the seafloor to the bottom of the hole (3058.5 mbsf). The relationship between porosity and strength was also derived from triaxial experiments on siltstones from the subducting Philippine Sea Plate under two end-member loading conditions of both triaxial extension and triaxial compression^32^. Then, they estimated the *in situ* formation strength from the V_p_ data using the above relations, assuming loading conditions of one-dimensional vertical consolidation in the forearc basin (~ 920 mbsf) and critical state loading in the accretionary prism (920–bottom of the hole). The difference between the strength derived from the V_p_-porosity-strength relation^13^ and the EST may arise because (1) the possible overconsolidation of sediments in the accretionary prism is not taken into account in the estimation; (2) the porosity calculated from V_p_ may be overestimated due to the time lag between the drilling operation and V_p_ measurement, as described above, which results in the estimated strength being lower than the EST; or (3) the EST may be overestimated because the EST is consistent with the triaxial strength of the sample under reverse-fault regime conditions^11^. It is difficult to identify the cause of the EST overestimation. One possibility is the effect of deformation rate on rock strength. As evidenced by laboratorial experiments, brittle strength is affected by strain rate; the higher strain rate is applied, the larger the strength becomes^34–36^. The experimental strength is generally determined at a displacement rate of ~ μm/s^2,9,11,31^,^33^. On the other hand, the displacement rate at a drill bit is ~ 0.3 m/s during the drilling in C0002P (12–1/4 in-diameter bit and 100 rotations-per-minute). The influence of the deformation rate on the strength of sedimentary rocks is insensitive compared to igneous rocks, however, it is reasonable to suppose that the effect is a cause of overestimation in the EST.

Theoretically, the relative value of the EST corresponds to the hardness of the drilled rock, and this is also supported by laboratory experimental data^37^. Hoberock and Bratcher (1996) suggested that the minimised drilling specific energy^15,20^ corresponding to the EST in this study can approximate the *in situ* compressive rock strength^37,38^. However, it cannot be assumed that the absolute value of the EST is equivalent to the rock strength under *in situ* stress conditions. The rock strength is generally measured by compression experiments in which differential stress is applied to a specimen assuming *in situ* stress conditions^34,39^. Although the EST is derived from drilling parameters that reflect the formation mechanical properties under *in situ* conditions, the *in situ* stress and pore pressure cannot be determined continuously, and hence, a verification experiment in the exact situation is not practical in principle. Thus, the EST at present should be treated as only an “equivalent strength”, which can be thought of as the relative *in situ* rock strength over a wide depth scale. The EST is obtained by stacking data from 5 m segments without the assumption of *in situ* stress conditions. Compared to laboratory experiments, which usually require intact specimens of several centimetres and supposition of the stress field, the EST has advantages of providing the relative *in situ* strength over a larger scale under *in situ* conditions, including the stress state, pore pressure and temperature. The large scale of the “drilling experiment” can reflect the representative formation strength. Numerous drilling experiments in the laboratory under various pressure and temperature conditions may allow determination of the correlation and lead to more accurate estimations of *in situ* formation rock strengths from the drilling parameters.

The EST profile shows characteristic positive peaks in Zone B (around 150–400 mbsf, 800–1000 mbsf, and some other depths) as well as larger amplitude fluctuations in Zones C and D. A comparison of the EST, carbonate content, and lithology at the site (Fig. 2) indicates that the local increases in the EST in Zone B may be due to strength hardening by carbonate materials. In this zone, the carbonate content is significantly higher (>20 wt%), which could make the sediments harder as a result of the cementing effect. In Zone C, the carbonate content profile shows a small peak at 1900–2000 mbsf, despite the absence of a significant spike in the EST at that depth. This could be due to the thinness of the carbonate layer, which may be too thin to affect the drilling parameters, or to the low strength contrast between the formation and carbonate layers. Sedimentological observations of cuttings did not indicate any remarkable cementation of formation in the interval^27,29^. This suggests that hardening due to cementation may control the strength in the forearc basin, but it is not significant in the accretionary prism.

Below lithological Unit IV ( > ~1000 mbsf), the EST profile shows larger fluctuations than the EST increases in the cementation zones. The fluctuation seems to correspond to the sand/mud ratio; the EST decreases in clay-rich intervals, likely indicating the baseline of the EST profile (e.g., around 1400 mbsf, 1800 mbsf, and 1950 mbsf).

One remarkable point is the absence of significant EST changes at the forearc basin–accretionary prism boundary (Fig. 2). This was also indicated in triaxial experiments on core samples^11^. The basin sediments overlying the accretionary sediments generally lie in a neutral stress state^40^; thus, the Kumano forearc sediments are expected to be consolidated in an isotropic manner, and the strength of the forearc sediments is expected to be smaller than that of the accretionary prism, which is older, deeper, and more consolidated by horizontal compression parallel to the subduction direction. Nevertheless, the baseline of the EST is roughly maintained at 20 MPa from 180 mbsf to 1410 mbsf across the major unit boundary and the recognised Zone B. Such strength contrast between the forearc basin and accretionary prism was not observed, likely because (1) carbonate cementation controls the strength baseline and (2) the horizontal compaction was not sufficient to strengthen the sediments in the shallow portion of the accretionary prism.

On the other hand, changes in the depth profile of the EST were observed in Zone C and around the Zone C–D boundary. The slope of the trend in Zone C (approximately 40 MPa/km) is steeper than that above 1442.5 mbsf (~20 MPa/km), and the EST maintains a high mean value (~60 MPa) in Zone D. The high EST value can be attributed to two possible effects: compaction by horizontal stress and/or diagenesis of the sediments. Common diagenetic processes in the shallow part of the accretionary prism, which consists mainly of detritus and sedimentary sand and mud, are albitisation, transitions of opal to quartz and smectite to illite, veining of quartz and carbonate minerals, and pressure solution^41^. Mineral measurements along Holes B, N, and P, however, do not show any significant increases in total clay or quartz abundance in the lower part of Zone B to Zone C^27^. Although the gradual transition of smectite to illite can be observed below ~2200 mbsf^42,43^, it does not explain the steeper EST increase in Zone C and the roughly stable profile in Zone D. Additionally, temperature estimation at site C0002 suggests that the *in situ* temperature does not exceed 100 °C, even at the bottom of the hole^44^, which is too low for other diagenetic processes. Structural observation of a spot core sample from 2163.0–2215.5 mbsf revealed that the bedding angle is almost vertical (~90°)^27^, indicating that the sediments were deformed mainly by horizontal compression. The folding of formations by compressive deformation at the shallower portion of the accretionary prism has been observed and identified by seismic images and LWD data^28,45–47^ Therefore, it is plausible that the increasing EST is caused by horizontal compression rather than mineral diagenesis. This interpretation does not mean that the sediments now experience such horizontal compression or suffer it at the present location; in fact, the accretionary prism lies in the strike faulting regime^5,12^.

The EST is a valuable geological-geophysical parameter because it is independent of other basic measurements, such as seismic reflection surveys and downhole logging, and is obtained without core samples or assumptions of the *in situ* environmental conditions. Although further study is necessary to assess the accuracy of the EST through laboratory drilling experiments, the EST analysis we presented here has significant advantage, particularly in non-core drilling or drilling in technically challenging areas, such as tectonically active margins and hydrothermal regions.

## Methods

### Definition of the equivalent strength (EST)

We proposed an evaluation of the depth profile of the equivalent strength (EST) to investigate continuous variations in the mechanical properties. Here, we define the EST based on the stress supplied to the formation assuming a simplified bit shape (see Supplementary Fig. S1) as:

1 $$EST[{\rm{Pa}}]=\frac{{F}_{ave}[{\rm{N}}]}{h[{\rm{m}}]\times (\frac{{R}_{1}}{2}-\frac{{R}_{2}}{2})[{\rm{m}}]}$$

where *F*_*ave*_ is the force at the cutter face when the formation brakes, *h* is the cutting depth, and *R*_1_ and *R*_2_ are the external and inner diameters of the drill bit, respectively. *F*_*ave*_ can be represented by the input torque measured at the surface (*T*_*m*_) and various unknown background torques (*T*_*b*_), and multiplying the right-hand side of equation (1) by the radius and integrating over the working face (*A*) gives:

2 $${\int }_{A}r\frac{{F}_{ave}}{h(\frac{{R}_{1}}{2}-\frac{{R}_{2}}{2})}dA={\int }_{\frac{{R}_{2}}{2}}^{\frac{{R}_{1}}{2}}r\frac{{F}_{ave}}{h(\frac{{R}_{1}}{2}-\frac{{R}_{2}}{2})}hdr=\frac{1}{2}{F}_{ave}(\frac{{R}_{1}}{2}+\frac{{R}_{2}}{2})={T}_{m}-{T}_{b}$$

Although it is impossible to measure or estimate *h* at any time from the recorded drilling parameters, the drilling advance per revolution (*ROP/RPM*) can represent the mean value of *h* for each rotation. Thus, the right-hand side of equation (1) can be rewritten using the recorded drilling parameters as:

3 $$EST[{\rm{Pa}}]=\frac{2({T}_{m}-{T}_{b})}{\frac{ROP}{RPM}\{{(\frac{{R}_{1}}{2})}^{2}-{(\frac{{R}_{2}}{2})}^{2}\}}$$

Strictly, the work of the weight on the bit (WOB) is also consumed to deform or break the formation at the bottom of the hole during drilling. If the drilled formation is weak enough and easily fragmented or deformed by vertical loading, the work of the WOB should be added next to the right-hand term of equation (3). However, the work of the WOB is generally negligible compared to the rotary work^15^. Equation (3) has the same style in principle as the mechanical specific energy (MSE) and the drillability strength of rock (D_s_) proposed by previous reports^15,34^, despite the different definitions and derivations. The EST represents a generalised form of these parameters, taking the background torque and inner diameter of a PCD bit.

It is difficult to measure or estimate *T*_*b*_ directly because it is a composite of torque losses, such as from friction between the pipe and formation, lateral acceleration, whirl, pipe torsion, fluid resistance, and so on. To eliminate these unknown toque losses and to estimate EST independently, equation (3) can be modified to provide a formula for the measured torque by the rate of penetration:

4 $$2{T}_{m}\times RPM=ROP\times \{{(\frac{{R}_{1}}{2})}^{2}-{(\frac{{R}_{2}}{2})}^{2}\}\times EST+2{T}_{b}\times RPM$$

In this equation, EST represents the gradient of the relationship between the measured torque energy per minute (left-hand term) and the volume rate of penetration. The stack of torque losses is shown by the intercept of the relation.

### Procedure of the EST calculation

Before applying the data collected at the C0002 holes to this equation, the data were resampled as mean values over 1 minute at every 10 seconds to reduce noise. Cross plots were created for each 5 m on the basis of the assumption that the formation properties are roughly homogeneous within the interval. If the mechanical properties are heterogeneous in the 5 m interval, the torque and the drilled volume will not be proportional, and equation (4) cannot be applied. In the ROP variation in the interval is small, a regression line cannot be determined, and the data cannot be used in the calculation. Therefore, we performed correlation analysis for each plot, and the EST and T_b_ were calculated when the correlation coefficient (*R*) was >0.4 and the *p*-value was <0.05 (5%). Examples of the torque-volume relations for the Nankai Trough drillings are shown in Supplementary Fig. S2.

The calculated EST profile (Fig. 2) fluctuates despite the above processing. To verify the cause of the variation, the validity of the EST is checked over the closed section at 2270–2275 mbsf, where the calculated EST is large (~122 MPa; Supplementary Fig. S3). In this 5-m section, two clusters are identified, and they follow each optimal fit line (Fig. S3a). These clusters are separated by 2272.1 mbsf. The trends in the logging data also change depending on the depth (Fig. S3b–e), indicating that the muddy (high gamma ray; GR, low resistivity, low V_p_) and easy-to-collapse (large calliper) formation gradually changes to a sandy and harder formation. The EST for the muddy (2270.0–2272.1 mbsf) and sandy (2272.1–2275.0 mbsf) regions was calculated as 25.0 MPa and 45.3 MPa, respectively. The clustering corresponds to the lithological boundary, and the EST values calculated from each cluster seem to correspond to each lithological feature. All data in this 5 m section were fitted with one line, satisfying the above significance criteria, and a very large EST was automatically calculated (black line in Fig. S3a). Thus, the EST includes variations due to section partitioning for automatic calculation. To avoid this problem, it is desirable to reduce the calculation interval to less than 5 m, shorter than the thickness of each lithofacies, for example, to several tens of centimetres. However, if the calculation interval is decreased to be applied to each lithology, the correlation line of each cluster cannot be obtained due to the small number of data points. Since 5 m is the minimum interval allowing sufficient correlation and to avoid artificial data selection from the Nankai data, we do not focus on each variance in the EST but use a moving average value for interpretation. By using more frequent data or more complex criteria for the lithology data, it would be possible to reduce variations in the EST.

## Electronic supplementary material

Supplementary materials
