# Supplementary material for: Continuous depth profile of the rock strength in the Nankai accretionary prism based on drilling performance parameters
Source: Sci Rep. 2018 Feb 14;8:2622. doi: 10.1038/s41598-018-20870-8 (PMC5813035; doi:10.1038/s41598-018-20870-8)
Supplement: Supplementary file 1 — Supplementary materials [file 41598_2018_20870_MOESM1_ESM.pdf]

Supplementary materials

Continuous depth profile of the rock strength in the Nankai accretionary prism based on drilling performance parameters

Yohei Hamada, Manami Kitamura, Yasuhiro Yamada, Yoshinori Sanada, Takamitsu Sugihara, Saneatsu Saito, Kyaw Moe, and Takehiro Hirose

Supplementary Figure S1:
Example drill bit and schematic image of cutting process.

Supplementary Figure S2:
Strategy of data conversion and *t*-test of accepted (a) and rejected datasets (b, c).

Supplementary Figure S3:
Data conversion diagram at 2270-2275 mbsf (a), and close up logging data (b–e) and drilling parameters (f–i) at the same section.

Supplementary Table S1
Calculation results of EST, background torque, and *p*-value of accepted data of all holes used in this study.

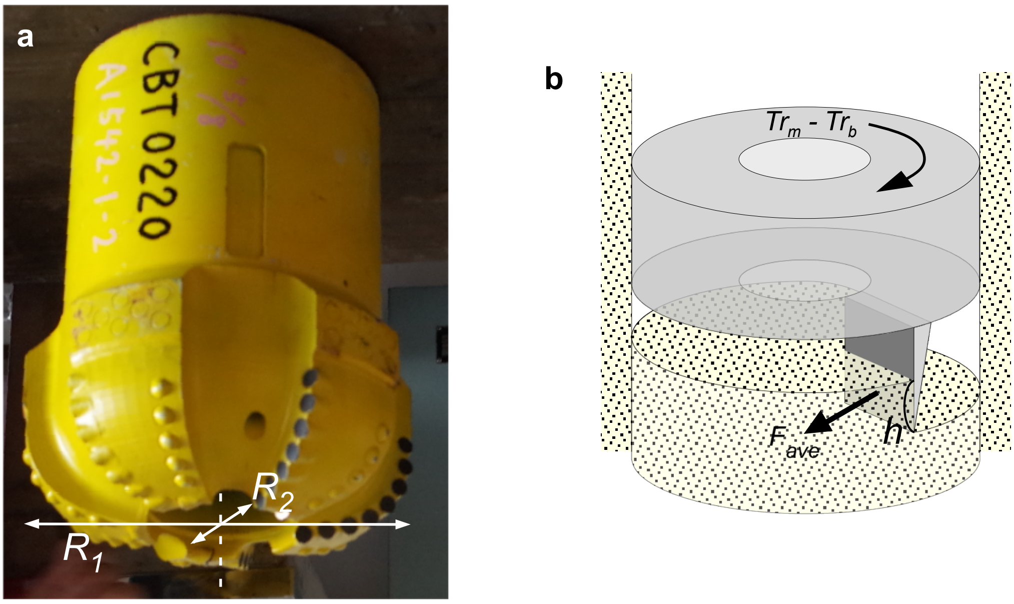

Figure S1
Example drill bit and schematic image of cutting process.
a: Photograph of a drill bit of the same type used for the C0002 drillings. The bit type is polycrystalline diamond compact (PDC), and the bit has a 10-5/8 inch outer diameter. **b**: Simplified force-balance model at the bit tip. The complex actual surface shape of the drill bit was converted into one work face. This simplification allows the surface-recorded drilling parameters to be applied to calculations of EST. Note that h does not always equal the designed cutter height.

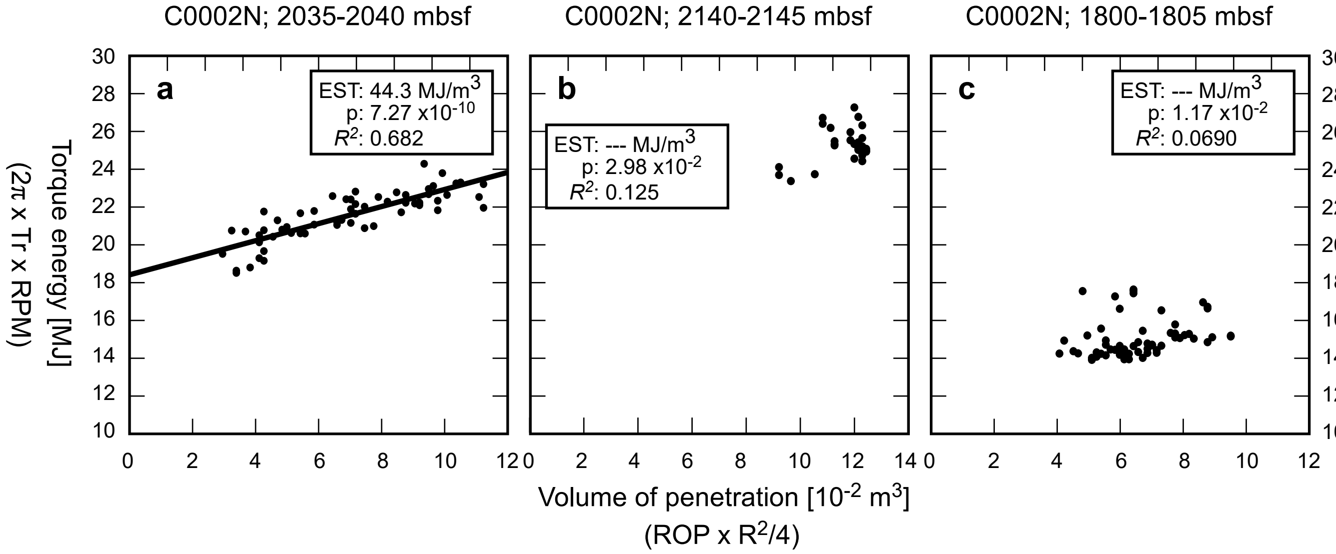

Figure S2

Strategy of data conversion and t-test of accepted (a) and rejected datasets (b, c).

a: Diagram of dataset at 2035-2040 mbsf in C0002N. Black dots represent substituted values of averaged drilling parameters at each 30-s interval into π x ROP x $R^2/4$ and 2π x TOR x RPM of equation (3). The diagram is clearly linear with a gradient of 4.43 x 10^6 and an intercept of 2.94 x 10^6, corresponding to the EST of 44.3 MPa and the background torque of 16.3 kN-m (mean RPM = 180.3 in this interval), respectively. **b** and **c**: Examples of rejected diagrams (2140-2145 mbsf and 1800-1805 mbsf in Hole N) because they do not meet the criteria due to lack of ROP variation and heterogeneity of formation. Note that the calculated R and p values of these two plots are not in the permitted range defined above. In these cases, EST estimations were not performed at the depths and invalid values were adopted.

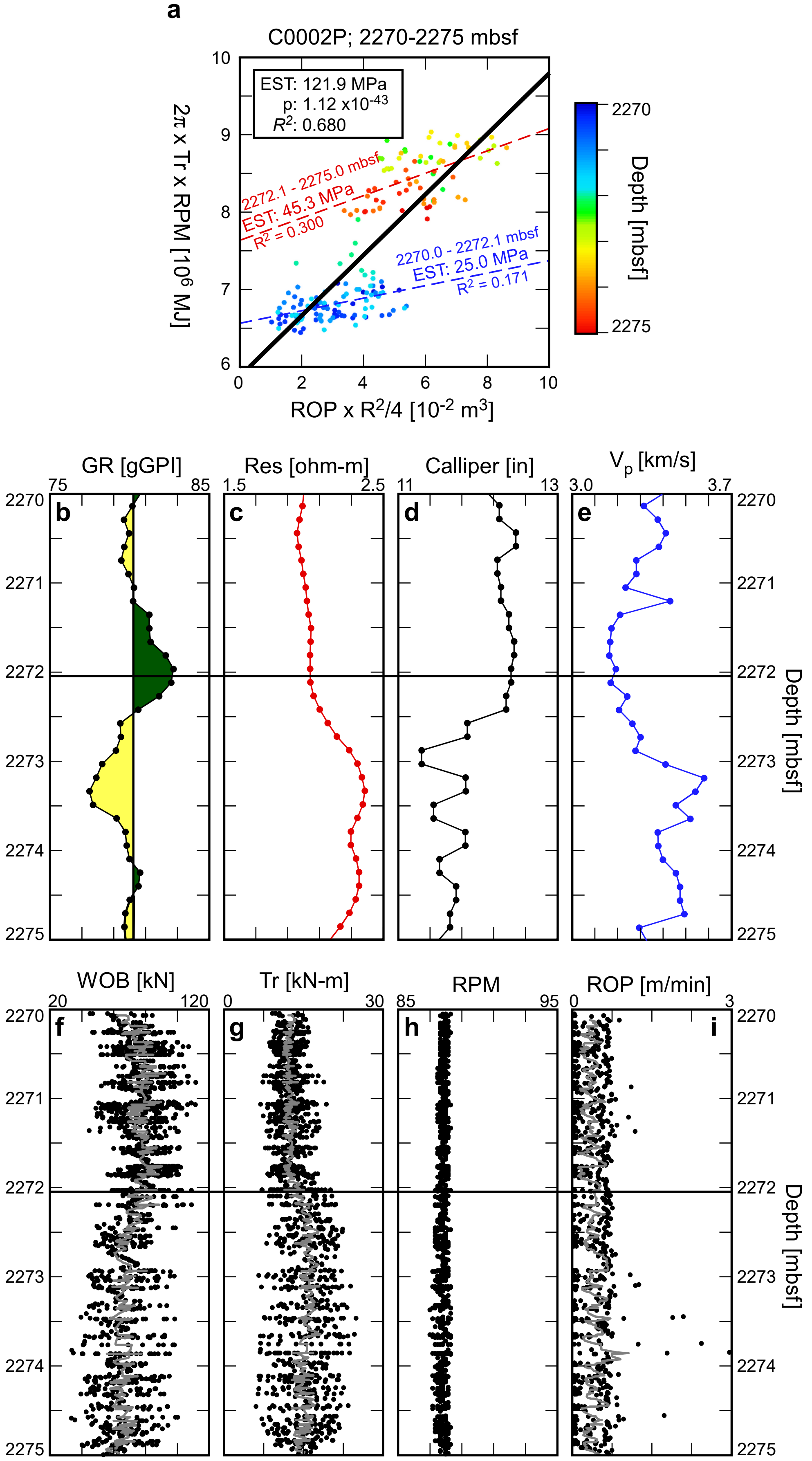

Figure S3

Data conversion diagram at 2270-2275 mbsf (a), and close up logging data (b - e) and drilling parameters (f - i) at the same section.

a: Diagram of data set at 2270-2275 mbsf in C0002P. Colors indicate the depth of each dot. **b-e**: Curves of downhole logging data. GR: Gamma ray, Res: Electric resistivity, Calliper: Borehole diameter: V_p: P-wave velocity. All measurements were conducted in Hole P (IODP, Exp. 348)[6]. f-i: Drilling parameters collected at the above interval.

Table S1

Hole H			
depth[mbsf]	EST [MJ]	Tb [kN-m]	p value
165.0	24.7	0.1	8.9E-03
180.0	11.9	2.0	7.0E-05
200.0	13.4	2.1	3.3E-02
215.0	14.9	2.7	4.4E-03
235.0	27.1	1.3	7.9E-03
240.0	39.4	0.2	2.4E-02
320.0	27.6	0.2	2.5E-05
330.0	42.2	0.4	2.3E-05
405.0	10.5	3.1	1.5E-02
470.0	34.8	0.8	1.3E-05
475.0	33.7	1.3	2.3E-12
500.0	14.6	2.3	1.3E-03
510.0	21.1	3.6	4.7E-04
515.0	11.5	3.8	6.9E-03
530.0	28.8	0.6	3.7E-02
550.0	27.3	2.0	2.4E-05
555.0	28.6	1.7	6.9E-03
560.0	19.4	3.0	2.9E-02
570.0	13.1	3.2	1.4E-02
635.0	31.3	2.5	4.4E-04
675.0	22.4	2.7	4.6E-04
710.0	16.7	3.6	2.9E-03
750.0	13.4	5.1	2.9E-02
755.0	38.2	1.4	3.6E-02
785.0	46.4	1.7	1.3E-03
790.0	17.5	4.5	1.4E-02
810.0	45.6	2.3	1.3E-02
815.0	56.3	2.8	7.0E-07
830.0	60.2	2.6	4.7E-03
885.0	44.6	1.8	7.9E-04
900.0	38.9	2.9	5.4E-06
925.0	50.2	2.8	5.0E-04
955.0	28.6	4.2	8.5E-03
1080.0	23.5	4.9	8.0E-04
1090.0	19.5	6.8	1.0E-02
1110.0	33.8	5.0	5.3E-06
1115.0	63.7	5.1	1.1E-11
1120.0	33.3	5.9	1.1E-03

Table S1 Calculation results of EST, background torque, and p-value of accepted data of all holes in this study.

Table S1

Hole I			
depth[mbsf]	EST [MJ]	Tb [kN-m]	p value
60.5	2.6	2.5	3.0E-02
65.5	0.2	3.3	3.6E-03
75.5	1.0	2.1	2.6E-03
80.5	2.8	0.8	8.5E-03
90.5	1.5	3.7	6.0E-03
95.5	3.7	2.3	5.1E-04
100.5	2.2	3.1	8.5E-04
135.5	7.9	1.6	1.6E-03
210.5	33.7	0.7	4.2E-04
215.5	25.1	2.7	4.5E-06
220.5	18.0	2.1	3.2E-02
240.5	11.6	5.3	3.3E-02
245.5	16.3	2.0	4.2E-03
330.5	10.3	3.2	8.6E-05
365.5	13.3	2.7	1.8E-03
395.5	12.9	3.6	4.0E-02
405.5	9.7	3.1	9.6E-07
435.5	2.0	4.9	4.1E-02
445.5	8.7	3.6	2.3E-06
480.5	12.7	3.4	5.6E-10
485.5	10.4	3.0	1.8E-06
520.5	10.7	2.9	3.8E-08
560.5	8.5	3.5	5.7E-08
595.5	17.2	2.8	1.3E-11
600.5	15.1	3.4	3.1E-09
625.5	5.4	5.8	2.2E-02
635.5	11.3	3.8	4.4E-05
640.5	14.1	3.8	1.6E-02
670.5	12.7	3.2	1.3E-03
675.5	7.7	4.2	7.2E-06
690.5	23.0	1.1	6.8E-04
700.5	11.0	3.8	3.3E-07
705.5	11.4	3.4	2.2E-02
750.5	10.3	3.6	2.8E-07
755.5	15.6	2.7	1.5E-03
785.5	13.5	3.8	4.1E-02
790.5	6.2	4.3	1.4E-06
830.5	7.5	4.7	2.4E-07
865.5	12.2	4.9	3.6E-08
875.5	27.0	2.2	6.8E-03
880.5	26.5	2.4	1.1E-05
895.5	40.9	2.1	5.4E-06
905.5	17.1	4.6	6.5E-08
920.5	26.1	5.7	4.8E-02
935.5	19.6	4.3	3.9E-04
940.5	17.2	4.6	3.5E-08
955.5	21.8	2.3	2.3E-04
960.5	23.3	0.9	6.7E-03
970.5	7.4	5.9	2.0E-04
975.5	21.1	3.6	3.4E-03
980.5	14.7	5.5	9.2E-04
1000.5	34.0	2.8	3.5E-03
1015.5	16.1	4.1	1.2E-04
1025.5	27.1	3.8	2.7E-03
1045.5	19.2	4.9	2.9E-04
1055.5	38.7	4.3	7.6E-05

Hole I (continued)			
depth[mbsf]	EST [MJ]	Tb [kN-m]	p value
1120.5	62.4	3.1	8.1E-10
1125.5	18.4	5.5	3.4E-02
1160.5	33.3	2.5	5.0E-04
1165.5	25.8	3.8	2.5E-07
1230.5	40.9	4.0	7.0E-03
1240.5	12.2	5.7	1.0E-02
1245.5	31.6	6.0	5.9E-04
1300.5	46.2	6.3	1.2E-10
1330.5	34.7	7.1	4.4E-06
1335.5	43.3	5.8	6.6E-06
1340.5	23.2	8.4	3.9E-02

Table S1

Hole J			
depth[mbsf]	EST [MJ]	Tb [kN-m]	p value
34.0	0.3	0.9	4.1E-05
39.0	0.3	0.9	8.1E-08
44.0	0.2	0.9	1.5E-03
49.0	0.4	0.5	1.4E-05
54.0	0.4	0.8	4.3E-09
59.0	0.4	0.5	2.1E-04
69.0	0.9	1.0	5.9E-03
79.0	0.4	1.2	1.7E-02
99.0	3.5	1.2	4.4E-02
104.0	4.1	2.1	7.1E-06
144.0	6.7	1.3	4.9E-09
179.0	12.5	1.6	2.6E-03
219.0	6.9	1.3	6.0E-04
249.0	7.9	2.4	4.3E-09
259.0	22.2	0.3	3.3E-05
294.0	20.1	0.2	1.8E-02
354.0	18.8	0.7	2.7E-02
414.0	9.9	1.6	7.3E-03
419.0	13.5	1.3	2.8E-02
434.0	21.0	0.4	8.0E-03
529.0	8.2	2.1	9.8E-03
544.0	23.0	0.6	5.0E-03
554.0	8.3	3.1	2.1E-02
564.0	17.4	1.8	2.3E-05
574.0	14.1	1.8	1.4E-02
604.0	10.2	1.9	4.8E-04
644.0	12.9	2.4	1.9E-02
679.0	18.5	0.6	8.1E-05
709.0	26.1	3.5	3.0E-03
749.0	16.4	1.3	6.3E-03
794.0	20.7	2.6	2.0E-04
824.0	25.3	1.6	3.0E-10
834.0	27.8	1.6	8.2E-07
864.0	21.8	1.9	2.4E-03
869.0	21.0	2.3	4.8E-05
874.0	29.5	1.1	6.3E-07
894.0	21.9	1.9	6.6E-06
899.0	30.0	1.8	3.2E-04

Hole K			
depth[mbsf]	EST [MJ]	Tb [kN-m]	p value
54.0	3.1	0.6	2.6E-03
59.0	0.7	1.4	3.3E-03
64.0	0.5	1.3	2.8E-02
69.0	0.3	1.4	7.7E-03
74.0	0.7	1.4	5.8E-05
79.0	0.6	1.3	2.9E-04
84.0	0.5	1.1	8.0E-05
94.0	1.1	1.4	5.2E-05
134.0	4.6	0.7	1.5E-04
169.0	4.3	1.3	2.4E-05
189.0	7.4	1.0	1.1E-02
204.0	4.2	2.1	1.4E-03
224.0	15.4	1.7	4.3E-04
264.0	7.4	2.2	4.5E-05

Table S1

Hole L			
depth[mbsf]	EST [MJ]	Tb [kN-m]	p value
144.0	7.7	1.1	4.1E-03
184.0	36.8	0.0	1.9E-04
264.0	32.0	0.9	6.3E-04
319.0	38.0	2.2	1.3E-08
334.0	18.6	3.4	1.3E-07
344.0	27.3	2.6	4.3E-04
439.0	26.7	2.4	1.8E-07
449.0	10.4	3.3	6.2E-04
454.0	21.1	2.8	1.2E-07
499.0	7.7	4.3	6.5E-04

Hole N			
depth[mbsf]	EST [MJ]	Tb [kN-m]	p value
782.5	3.5	5.0	5.6E-03
787.5	5.1	6.3	9.6E-05
792.5	12.1	4.2	2.2E-11
797.5	10.2	4.0	1.3E-09
807.5	6.8	5.4	2.8E-03
812.5	6.9	5.6	4.0E-02
817.5	13.1	6.0	1.1E-02
827.5	13.6	7.4	3.2E-04
837.5	20.0	7.5	2.2E-06
862.5	74.5	3.9	9.1E-39
867.5	50.1	4.2	5.9E-16
872.5	29.0	4.2	2.4E-12
902.5	6.6	4.1	4.2E-03
907.5	18.0	4.0	1.9E-05
952.5	5.6	4.5	1.3E-32
957.5	7.3	4.1	2.8E-05
962.5	20.3	2.4	5.0E-04
967.5	9.7	3.9	4.7E-07
982.5	19.2	5.3	1.4E-04
987.5	19.0	5.4	3.8E-10
992.5	29.5	4.7	1.5E-07
997.5	40.6	4.4	6.0E-05
1022.5	28.6	5.7	4.0E-06
1027.5	18.1	5.9	1.5E-03
1052.5	11.9	6.4	4.4E-03
1067.5	16.9	5.9	4.2E-05
1072.5	8.8	7.0	8.9E-03
1077.5	19.6	6.5	3.1E-03
1082.5	11.4	6.3	2.2E-03
1092.5	10.1	7.0	8.6E-03
1097.5	14.3	6.8	3.5E-04
1112.5	11.4	7.5	4.5E-02
1117.5	12.0	7.4	3.3E-05
1122.5	8.0	7.5	2.7E-02
1137.5	17.3	7.5	8.8E-08
1142.5	17.1	6.9	1.3E-04
1147.5	14.3	7.2	4.8E-10
1157.5	21.2	7.8	1.3E-05
1167.5	13.0	8.5	3.8E-02
1172.5	9.6	9.2	2.7E-02
1177.5	18.0	8.0	4.8E-12
1182.5	12.7	8.5	3.9E-04
1202.5	21.6	7.8	7.5E-07
1207.5	38.9	7.0	4.6E-07
1212.5	24.0	8.7	1.4E-03
1222.5	12.3	9.2	5.1E-04
1227.5	15.1	8.9	8.8E-08
1237.5	39.7	7.0	1.1E-05
1252.5	9.5	10.5	8.8E-03
1257.5	20.7	9.4	1.7E-03
1267.5	11.6	10.8	3.5E-03
1272.5	31.4	9.6	1.2E-10
1277.5	30.4	8.6	8.9E-09
1282.5	33.6	9.6	1.6E-08
1287.5	25.1	9.5	5.7E-10
1297.5	38.4	7.7	3.7E-04

Table S1

Hole N (continued)			
depth[mbsf]	EST [MJ]	Tb [kN-m]	p value
1302.5	29.1	8.2	4.6E-07
1332.5	21.2	8.3	3.2E-05
1337.5	8.2	9.7	1.2E-02
1342.5	10.4	9.6	3.8E-05
1347.5	6.0	10.2	1.8E-02
1357.5	16.1	9.8	3.2E-02
1367.5	14.5	10.2	7.7E-03
1372.5	14.5	9.7	1.5E-03
1407.5	21.8	8.5	9.1E-06
1412.5	5.6	9.6	5.4E-03
1427.5	18.8	10.6	3.8E-03
1437.5	8.5	11.7	6.5E-04
1447.5	42.1	8.5	2.7E-08
1487.5	27.4	9.5	6.0E-05
1492.5	22.0	11.8	7.6E-03
1522.5	60.0	8.7	1.1E-08
1527.5	23.4	11.2	1.2E-03
1537.5	30.7	11.4	3.0E-06
1542.5	13.6	12.4	2.5E-03
1547.5	27.8	11.0	3.4E-03
1557.5	34.6	10.3	2.2E-02
1562.5	24.7	11.6	6.2E-08
1572.5	32.4	13.6	2.8E-03
1577.5	44.0	12.2	6.7E-04
1582.5	13.5	13.7	3.6E-03
1587.5	36.9	12.4	8.8E-06
1592.5	34.6	12.5	1.9E-07
1602.5	35.2	11.0	1.1E-09
1607.5	29.1	12.3	9.8E-05
1612.5	55.6	9.9	5.3E-08
1617.5	20.2	13.3	1.9E-02
1632.5	63.0	13.1	1.6E-05
1662.5	47.8	11.1	1.8E-18
1667.5	18.8	13.1	7.8E-07
1672.5	25.8	12.1	7.7E-05
1677.5	17.3	12.3	5.7E-04
1682.5	30.2	12.6	4.7E-05
1687.5	34.5	12.5	5.6E-07
1692.5	74.2	10.5	1.0E-16
1697.5	20.2	13.6	9.5E-03
1702.5	29.4	12.2	1.4E-06
1707.5	43.4	11.6	5.9E-09
1727.5	55.5	11.1	4.8E-04
1732.5	15.9	12.7	3.5E-03
1747.5	34.7	12.0	2.6E-05
1752.5	30.9	11.4	9.1E-08
1767.5	28.7	12.6	1.8E-04
1772.5	31.1	12.5	1.4E-05
1777.5	20.9	12.7	8.8E-05
1782.5	12.2	12.8	5.9E-03
1787.5	30.3	11.8	6.5E-09
1792.5	23.7	12.9	6.8E-06
1797.5	22.2	12.5	3.3E-08
1802.5	25.4	13.2	1.2E-02
1817.5	10.8	13.4	1.9E-03
1822.5	10.5	12.9	9.6E-03

Hole N (continued)			
depth[mbsf]	EST [MJ]	Tb [kN-m]	p value
1827.5	42.9	11.1	4.2E-10
1842.5	27.3	13.0	2.6E-06
1847.5	57.9	11.7	8.2E-08
1852.5	45.2	12.7	2.3E-07
1857.5	43.2	12.1	1.8E-05
1862.5	48.4	12.2	6.7E-11
1867.5	19.8	14.2	4.8E-02
1872.5	16.2	14.6	2.5E-03
1877.5	17.1	14.9	9.3E-03
1882.5	76.2	12.1	1.1E-09
1887.5	24.8	14.2	1.0E-02
1892.5	48.0	12.8	9.3E-07
1907.5	31.4	15.5	9.7E-04
1912.5	37.5	15.5	9.3E-06
1927.5	21.6	16.2	2.6E-02
1937.5	13.8	16.0	1.2E-03
1947.5	25.4	15.1	1.7E-05
1962.5	28.0	15.4	1.2E-03
1967.5	34.0	15.3	2.3E-07
1982.5	43.9	14.7	9.4E-06
1987.5	37.2	14.3	1.9E-05
1997.5	47.6	14.5	6.7E-06
2002.5	21.9	15.5	3.3E-02
2012.5	56.6	14.9	1.2E-08
2017.5	74.0	14.2	2.2E-13
2022.5	87.8	14.3	6.6E-14
2027.5	27.5	17.6	1.6E-04
2032.5	49.5	16.5	7.3E-10
2037.5	44.3	16.3	4.7E-16
2042.5	79.2	14.3	1.1E-09
2047.5	49.6	15.5	1.1E-05
2052.5	62.1	14.2	8.0E-11
2057.5	16.8	19.7	2.7E-02
2067.5	26.0	17.8	6.2E-04
2072.5	17.7	18.1	6.8E-03
2082.5	23.9	19.2	2.9E-02
2087.5	44.8	15.8	3.2E-09
2097.5	46.4	17.0	3.6E-06
2102.5	47.4	15.6	5.3E-13
2112.5	61.4	15.2	1.1E-09
2122.5	42.9	16.1	1.7E-07
2127.5	33.3	16.9	5.6E-08
2132.5	71.2	15.0	7.9E-09
2142.5	34.0	18.8	3.0E-02
2162.5	76.6	17.1	1.6E-15
2172.5	40.4	17.5	1.1E-11
2182.5	38.5	18.2	1.5E-06
2192.5	62.5	18.2	4.9E-14
2207.5	63.8	19.1	2.3E-18
2212.5	41.8	21.8	6.3E-04
2222.5	41.2	20.1	1.1E-07
2227.5	30.5	21.7	8.7E-03
2232.5	68.0	18.3	1.4E-13
2237.5	48.3	19.8	2.4E-10
2242.5	72.8	17.8	4.4E-20
2247.5	72.3	17.4	1.6E-08

Hole N (continued)			
depth[mbsf]	EST [MJ]	Tb [kN-m]	p value
2252.5	88.2	16.6	1.0E-16
2257.5	53.2	20.7	6.6E-22
2262.5	40.3	20.1	3.8E-04
2272.5	67.3	18.8	1.0E-08
2277.5	86.8	16.5	2.3E-14
2287.5	48.8	18.6	2.1E-06
2292.5	80.0	15.7	1.4E-12
2307.5	67.2	20.6	9.6E-11
2312.5	85.5	17.6	5.0E-06
2317.5	29.9	22.6	1.1E-03
2327.5	28.3	21.9	4.0E-03

Hole P			
depth[mbsf]	EST [MJ]	Tb [kN-m]	p value
1982.5	27.2	12.4	9.2E-04
1987.5	28.6	13.2	2.3E-03
2042.5	29.3	12.2	3.2E-06
2062.5	16.4	15.1	4.6E-04
2087.5	26.5	12.3	4.5E-06
2102.5	41.6	11.1	1.3E-02
2142.5	53.2	12.4	4.2E-04
2182.5	78.7	10.6	9.3E-22
2187.5	102.1	10.8	1.8E-48
2192.5	71.4	11.1	1.0E-23
2197.5	89.2	11.3	4.0E-21
2207.5	112.5	10.3	3.2E-67
2227.5	45.7	12.5	6.6E-20
2232.5	22.7	13.1	7.2E-08
2237.5	28.0	13.9	3.2E-16
2242.5	59.3	12.8	3.6E-12
2257.5	33.2	12.8	2.9E-11
2267.5	39.9	12.2	1.9E-19
2272.5	121.9	10.7	1.1E-43
2277.5	32.6	13.2	3.9E-10
2282.5	29.3	12.6	4.6E-18
2292.5	27.7	13.0	1.2E-13
2297.5	42.4	13.6	1.7E-07
2302.5	43.3	12.8	3.1E-12
2307.5	95.9	11.7	4.8E-34
2312.5	38.3	15.5	3.4E-05
2317.5	85.3	12.0	1.9E-13
2322.5	27.7	14.0	2.6E-11
2332.5	32.1	14.4	3.2E-08
2337.5	78.2	12.9	9.7E-19
2342.5	44.3	15.0	2.5E-10
2347.5	56.3	13.7	1.8E-16
2352.5	79.1	12.4	1.4E-27
2357.5	67.6	12.2	7.3E-26
2362.5	64.7	13.3	9.9E-12
2367.5	40.7	14.7	2.4E-09
2372.5	39.3	14.3	1.7E-06
2377.5	29.5	13.6	2.4E-07
2382.5	98.4	11.8	7.6E-23
2392.5	69.3	12.6	3.8E-26
2397.5	46.6	13.0	3.9E-14
2407.5	74.3	12.7	4.6E-16
2412.5	84.6	12.6	1.6E-21
2417.5	49.9	14.0	3.2E-09
2422.5	75.6	13.2	1.5E-09
2432.5	39.0	13.8	1.3E-08
2437.5	59.5	12.9	1.8E-13
2442.5	22.2	13.9	5.5E-08
2447.5	55.6	13.7	4.0E-13
2452.5	26.6	14.6	3.6E-07
2457.5	45.9	14.1	7.2E-08
2462.5	31.1	14.5	2.7E-13
2467.5	66.2	13.3	2.0E-17
2472.5	61.7	14.0	1.5E-15
2477.5	33.8	14.5	4.0E-09
2487.5	20.8	14.5	5.2E-08

Table S1

Hole P (continued)			
depth[mbsf]	EST [MJ]	Tb [kN-m]	p value
2492.5	70.6	14.0	7.2E-14
2497.5	69.5	13.7	2.2E-06
2502.5	46.3	14.5	2.4E-17
2507.5	60.5	13.9	1.0E-11
2512.5	72.5	14.3	9.8E-11
2517.5	86.2	13.6	1.2E-10
2522.5	23.4	15.5	3.0E-06
2527.5	53.3	15.0	1.2E-05
2537.5	44.6	15.6	2.2E-07
2552.5	74.2	14.0	8.4E-16
2557.5	42.8	16.4	2.1E-05
2562.5	47.9	15.4	1.9E-07
2577.5	33.6	14.9	1.8E-07
2582.5	38.1	15.3	8.1E-16
2587.5	54.5	15.0	1.1E-17
2592.5	41.7	14.8	3.2E-12
2597.5	24.3	15.4	1.9E-09
2607.5	56.3	16.3	1.7E-10
2617.5	51.2	16.0	3.9E-05
2622.5	100.4	13.8	3.9E-07
2627.5	69.8	15.7	5.0E-08
2642.5	97.1	14.3	7.5E-13
2647.5	50.2	14.5	1.6E-07
2652.5	36.2	15.7	1.1E-07
2657.5	57.2	14.7	7.8E-18
2662.5	115.0	13.2	4.0E-11
2667.5	80.1	14.4	8.9E-10
2672.5	39.7	15.4	6.0E-06
2682.5	41.9	15.8	2.5E-06
2687.5	46.8	15.7	1.8E-05
2717.5	84.2	15.0	8.7E-11
2722.5	56.1	16.1	5.8E-08
2727.5	39.5	16.8	3.8E-10
2732.5	49.8	16.4	1.3E-05
2737.5	27.3	17.7	7.7E-05
2742.5	48.7	16.3	1.7E-09
2777.5	61.2	15.8	1.3E-09
2792.5	40.2	17.4	5.4E-06
2827.5	36.2	16.7	1.6E-08
2832.5	29.7	16.3	2.8E-12
2837.5	28.5	16.9	2.8E-08
2842.5	56.4	16.5	3.5E-14
2847.5	72.7	15.9	3.3E-09
2852.5	33.0	17.6	1.2E-08
2857.5	66.7	16.7	1.1E-10
2862.5	46.4	17.6	1.8E-05
2867.5	55.9	17.1	5.0E-17
2872.5	115.9	14.8	1.0E-11
2892.5	96.2	15.9	8.6E-07
2907.5	70.6	17.0	4.3E-08
2912.5	93.1	16.1	1.5E-04
2942.5	125.9	15.6	2.7E-16
2947.5	85.4	17.3	2.0E-13
2957.5	87.4	17.0	1.9E-11
2972.5	66.9	17.5	3.6E-05
2982.5	88.8	17.5	1.1E-09

Hole P (continued)			
depth[mbsf]	EST [MJ]	Tb [kN-m]	p value
2997.5	112.5	15.7	1.0E-09
3007.5	47.6	17.9	6.8E-05
3012.5	74.1	17.5	2.1E-10
3017.5	53.4	17.9	3.4E-08
3022.5	105.5	17.2	2.7E-06
3037.5	107.8	16.9	1.8E-08
